# *Plasmodium* spp. and *Borrelia burgdorferi* co‐infection associated with antiphospholipid syndrome in a returned traveler: a case report

**DOI:** 10.1002/ccr3.871

**Published:** 2017-03-02

**Authors:** Nélia Neves, André Silva‐Pinto, Helena Rocha, Susana Silva, Edite Pereira, Antonio Sarmento, Lurdes Santos

**Affiliations:** ^1^ Infectious Diseases DepartmentCentro Hospitalar São JoãoPortoPortugal; ^2^ Infectious Diseases DepartmentFaculty of Medicine of University of PortoPortoPortugal; ^3^ Neurology DepartmentCentro Hospitalar São JoãoPortoPortugal; ^4^ Neurosciences and Mental Health DepartmentFaculty of Medicine of University of PortoPortoPortugal; ^5^ Internal Medicine DepartmentCentro Hospitalar São JoãoPortoPortugal; ^6^ Institute of Health Innovation and Investigation (I3S)Nephrology and Infectious Diseases GroupNational Institute of Biomedical EngineeringPortoPortugal

**Keywords:** Antiphospholipid syndrome, lyme disease, malaria, neuroborreliosis, *Plasmodium*

## Abstract

The differential diagnosis of fever in a returned traveler is wide and challenging. We present a case of a patient working in Africa, who returned with fever, constitutional symptoms, headache, and blurred vision. An initial diagnosis of malaria was made, and additional workup revealed *Borrelia burgdorferi* co‐infection and antiphospholipid syndrome.

## Introduction

In a febrile patient returning from a tropical region, malaria is one of the diagnoses an attending clinician must consider 1. However, when a patient with a confirmed diagnosis of malaria has an unexpected clinical evolution, co‐infections should be considered.

In patients with Lyme disease, borreliosis co‐infection is common. The most common co‐infections in these patients are Babesia (32%), Bartonella (28%), Ehrlichia (15%), Mycoplasma (15%), Rocky Mountain spotted fever (6%), Anaplasma (5%), and tularemia (1%) 2. Concomitant infection by *B. burgdorferi* and *B. microti* (an intraerythrocytic parasite distantly related to *Plasmodium* spp.), both tick‐borne diseases, may be associated with an increase in the severity of the disease 3. There are also several case reports of Plasmodium co‐infections with other agents 4.

However, co‐infection of malaria with *Borrelia* spp. is rare. Few reports of *Plasmodium* spp. co‐infection with *Borrelia recurrentis* have been reported in the English literature 5, 6, 7.

As far as we are aware, we are writing the first report of *Plasmodium* spp. and *Borrelia burgdorferi* co‐infection (a co‐infection of a tropical parasite and a nontropical bacterium).

## Case History

A 42‐year‐old Caucasian man was transferred from a district hospital to our Infectious Disease Department due to fever (38–39°C), asthenia, myalgia, headache, and blurred vision, 7 days after having returned from Angola.

He had an unremarkable past medical history except for arterial hypertension for which he was taking olmesartan/hydrochlorothiazide (10/6.25 mg id) and nebivolol (2.5 mg id). He did not have past or current smoking habits, and he was not obese.

The patient was originally from a rural town in the north of Portugal, and he had been living in Angola for the past 4 years, working as a welder. During his stay in Angola, he traveled to Portugal on a regular basis (three to four times a year). He lived in different provinces in Angola, including Lunda Norte, Lunda Sul, Moxico, Cuando Cubango, and Luanda. He reported that he received immunizations before traveling and took antimalarial prophylaxis during his first year in Angola (mefloquine 250 mg once a week). Four months before traveling to Portugal, he was diagnosed with malaria in Angola and treated with an outpatient regimen he could not recall. No other important epidemiological history was reported.

Two months before admission, he started to complain of increasing asthenia and recurrent episodes of low‐grade fever associated with myalgia and nonspecific episodes of visual changes that the patient did not specify well. He did not seek medical attention at this time. Seven days before admission, he returned from Angola on holiday and, during the flight, he complained of blurred vision, mild frontal headache, worsening of asthenia, and generalized myalgia. He started experiencing daily episodes of chills and spiking fever (39°C) with persistent blurred vision.

Five days before admission, he was seen in a district hospital. At that time, the patient appeared to be well and the blood analysis was within the reference values except for C‐reactive protein (CRP), which was 100.3 mg/L (Table 1). The peripheral blood smear was negative for *Plasmodium*. A head computerized tomography (CT) scan revealed signs of maxillary sinusitis, and the patient was referred to an ear‐nose‐throat specialist. The patient was treated with amoxicillin–clavulanate for acute bacterial sinusitis and was discharged. Three days later, he returned to the hospital; the peripheral blood smear was negative for *Plasmodium* (Table 1), and the patient was discharged again.

**Table 1:** Patient's laboratory data

Variable	Reference range	Five days before admission (district hospital emergency room)	Two days before admission (district hospital emergency room)	On admission to the district hospital	Admission to our hospital
Hemoglobin (g/dL)	13.0–18.0 (men)	15.9	13.6	11.2	8.7
Hematocrit (%)	43–55	–	40.6	33.6	25.3
Erythrocyte count (x10^9^/L)	4.4–6.0	–	4.7	3.9	3.13
Reticulocytes (%)	0.5–2.5	–	–	–	1
Erythrocyte sedimentation rate (mm/hr)	1–7	–	–	–	50
White‐cell count (x10^9^/L)	4.0–11.0	3.5	4.46	4.97	4.66
Neutrophils (%)	53.8–69.8	–	82.1	57.5	71.0
Lymphocytes (%)	22.6–36.6	–	6.74	28.2	18
Monocytes (%)	4.7–9.7	–	9.64	7.87	9.9
Eosinophils (%)	0.6–4.6	–	0.73	5.88	0.0
Basophils (%)	0.0–1.5	–	0.76	0.55	0.2
Platelet count (x10^9^/L)	150–400	131	40	31	65
Prothrombin time (seconds)	10.2–13.9	–	–	–	12.8
Partial thromboplastin time (seconds)	24.5–36.5	–	–	–	48.1
Albumin (g/L)	38–51	–	–		29.5
Aspartate aminotransferase (U/L)	10–37	–	–	24	48
Alanine aminotransferase (U/L)	10–37	–	–	45	48
γ‐glutamyltransferase (U/L)	10–49	–	–	123	69
Total bilirubin (mg/dL)	<1.2	–	–	1.1	1.33
Direct bilirubin (mg/dL)	<0.4	–	–	0.29	0.46
Lactate dehydrogenase (U/L)	135–225	–	–	619	580
Phosphorus	2.7–4.5	–	–	–	1.6
Magnesium (mEq/L)	1.55–2.05	–	–	–	1.48
Urea nitrogen (mg/dL)	10–50	–	–	59	31
Creatinine (mg/dL)	0.67–1.17	–	–	1.06	0.65
Creatine kinase, (U/L)	10–172	–	–	31	36
Myoglobin (ng/mL)	<146.9	–	–	32	26.9
C‐reactive protein (mg/L)	<3.0	103.0	114.9	353.7	224.2
Peripheral smear	Normal	Negative for *Plasmodium* spp.	Negative for *Plasmodium* spp.	*Plasmodium* spp.	*Plasmodium* spp. (<0.1%)

He went back to the district hospital for the third time and was admitted to the internal medicine ward. On the day of admission, the platelet count was 31 × 10^9^/L and the CRP was 353.7 mg/L. The peripheral blood smear was positive for *Plasmodium* spp., and antimalarial therapy with oral quinine and doxycycline was started; arterial blood gas (ABG) revealed respiratory alkalosis with hyperlactacidemia (4.0 mmol/L for a reference range: <1.0 mmol/L). The D‐dimer assay was also elevated (4.50 *μ*g/mL), and a thoracic CT scan showed a mild pulmonary embolism affecting the upper segmental branches of the middle lobe artery. On the second day of admission at the district hospital, the patient developed an altered mental status with increased lethargy and was transferred to our hospital.

When the patient arrived to our department, he was pale, dehydrated, and icteric. He had a temperature of 37°C, blood pressure of 150/110 mmHg, a heart rate of 111 beats per minute, a respiratory rate of 22 breaths per minute, and an oxygen saturation of 100% at room air, with normal chest auscultation; the abdomen was soft, and hepatosplenomegaly was noted. The patient was awake during the neurological examination but showed signs of confusion, disorientation, and marked cognitive slowing. A slight left central facial palsy was described, with no other cranial neuropathies. He had brisk reflexes, with preserved muscular strength and sensory modalities. No movement disorders or involuntary or abnormal postures were noticed.

The hemoglobin level was 8.7 g/dL. The other laboratory results are shown in Table 1. The rapid diagnostic testing (RDT) for malaria (BinaxNOW^®^) was positive for histidine‐rich protein II and pan‐malarial antigen (aldolase). The examination of the thin blood smear confirmed an infection with *Plasmodium spp.,* and parasitemia was estimated <0.1%. The molecular biology test (polymerase chain reaction (PCR)) was positive for *Plasmodium falciparum*. The NS1 antigen and antibody testing for dengue was negative (SD BIOLINE Dengue Duo^®^).

Blood cultures were collected. Testing for human immunodeficiency virus and hepatitis C virus antibodies was negative, as was hepatitis B virus surface antigen.

Chest radiography revealed a bilateral pulmonary infiltrate. Urinary antigens for *Streptococcus pneumonia* and *Legionella pneumophila* were negative. The abdominal ultrasound examination showed hepatosplenomegaly without abdominal lesions. The head CT scan showed signs of sinusitis without brain lesions or leptomeningeal enhancement.

The patient was admitted to the infectious diseases intensive/intermediate care unit (ICU). Antimalarial treatment with intravenous (IV) quinine 600 mg q8 h and IV doxycycline 100 mg q12 h was initiated. Anticoagulation with low‐molecular‐weight heparin was started for pulmonary embolism. Neurology and ophthalmology consultations were requested.

**Figure 1:**
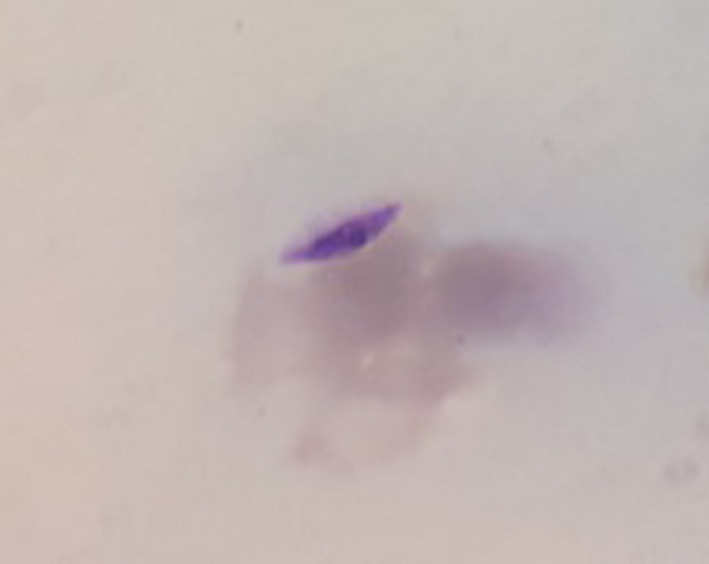
Patient's bone marrow shows spindle‐shaped immature Plasmodium falciparum gametocytes.

On the first day at the ICU, a lumbar puncture was performed, which revealed a lymphocytic pleocytosis (14 cells), elevated protein concentration (1 g/L), and normal glucose concentration (81 mg/dL); a bone marrow aspirate was also performed as part of the investigation which revealed *Plasmodium falciparum* gametocytes (Fig. 1), without any other findings. The bacteriology and mycobacteriology examinations of cerebrospinal fluid (CSF) were negative; the parasitological examination for *Trypanosoma brucei* detection was negative in CSF and blood. CSF‐PCR for *Borrelia burgdorferi* “sensu lato” was positive (in‐house technique with external quality control), with a negative CSF *Borrelia* spp. antibody. The serology for *Borrelia* spp. was performed, and both IgM and IgG were positive (screened by ELISA and confirmed by Western blotting). The oligoclonal bands were positive in CSF and in the blood. The diagnosis of neuroborreliosis was assumed, and IV ceftriaxone 2 g q12 h was added to the therapeutic regimen for 14 days.

**Figure 2:**
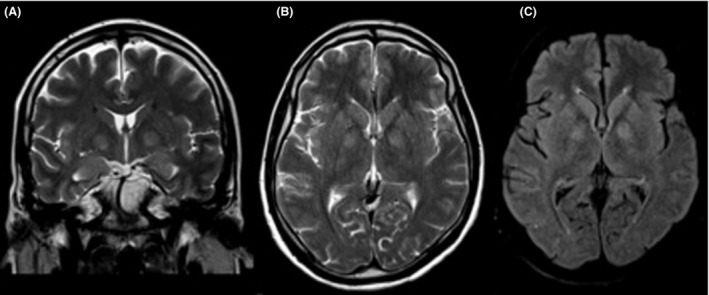
Patient's brain MRI reveals signal abnormalities in basal ganglia, with no mass effect in (A) coronal T2 TSE, (B) axial T2 TSE, and (C) axial T2 FLAIR.

The brain magnetic resonance imaging (MRI) revealed signal abnormalities in the T2/FLAIR sequences in the basal ganglia, without mass effect, gadolinium enhancement, or restriction in the DWI sequences, most probably suggestive of a metabolic etiology. There were no pathological parenchyma abnormalities (Fig. 2).

The clinical condition of the patient progressively improved with remission of fever, myalgia, headache, and focal neurological deficits. Parasitemia was cleared on day 2, and blood cultures remained sterile.

In order to clarify the occurrence of a pulmonary embolism, apparently unrelated with malaria and neuroborreliosis infection, autoimmunity investigation was performed, which revealed positive IgM anticardiolipin (90.6 MPL; reference: <15), IgM anti‐beta 2 glycoprotein 1 (18.9 SMU; reference: <15), and positive lupus anticoagulant antibodies (coagulometric method), suggestive of antiphospholipid syndrome. Levels of antithrombin and C and S proteins were 0.69 U/mL (reference: 0.70–1.35), 0.59 U/mL (reference: 0.75–1.25), and 0.50 U/mL (reference: 0.60–1.40), respectively. The tests for antinuclear antibodies (ANA), anti‐double‐stranded DNA (dsDNA) antibody, antineutrophil cytoplasmatic antibodies (ANCA), rheumatoid factor, neuronal antibodies, and anti‐extractable nuclear antigen antibodies (ENA) were negative.

The patient was discharged on day 29, asymptomatic, under oral anticoagulation.

Nine days after discharge from our hospital, he initiated high fever and generalized myalgia. After 4 days of symptoms, he came to our emergency room. Physical examination showed fever, slight polypnea (24 breaths/min), but no other abnormalities.

The analyses showed pancytopenia (hemoglobin 9.8g/dL, leukocytes 1180/*μ*L, and platelets 54,000/*μ*L) and elevated C‐reactive protein (202 mg/L). There were no abnormalities in the hepatic and renal functions, but the LDH was high (534 IU/L). The blood smear showed 0.6% *Plasmodium* spp. parasitemia, and the blood antigen rapid test was positive for pan‐malarial antigen and negative for histidine‐rich protein (BinaxNOW^®^). It was not possible to perform the PCR technique.

The patient was treated with oral quinine and doxycycline for 7 days. There was a gradual improvement in his clinical status and laboratorial tests, and he was discharged after 7 days. The glycogen‐6‐phosphate dehydrogenase (G6PD) test was negative, and he was treated with primaquine 15 mg id for 14 days. He was re‐evaluated in the outpatient clinic 4 weeks after being discharged, and he was asymptomatic with normal blood test analysis.

Four months after the neuroborreliosis treatment, the serologic test for *Borrelia burgdorferi* sensu lato was still positive (both IgM and IgG), as was the lupus anticoagulant antibodies.

The brain MRI was repeated 1 year after discharge. It showed a complete resolution of the basal ganglia signal abnormalities previously reported, arguing for a metabolic and transient cause, as expected. Repeated autoimmunity studies revealed negative anticardiolipin and anti‐beta 2 glycoprotein 1 (1.2 MPL and 6.3 SMU, respectively) after 4 months and negative antilupus anticoagulant antibody after 1 year (the lupus anticoagulant was still positive at 4 months). The anticoagulation was stopped after 1 year. There were no other thrombotic phenomena during the follow‐up.

## Discussion

*Plasmodium* spp. and *Borrelia* spp. co‐infection is an uncommon condition described in travelers. The only co‐infections reported in the literature are caused by *Borrelia recurrentis* 6, 7. In this case, the patient acquired malaria in West Africa (during his stay in Angola) and was probably infected with *Borrelia burgdorferi* sensu lato in Europe (while he was visiting his family in the north of Portugal).

The diagnosis of malaria was initially suspected in this patient as it is still the most common cause of fever in returned travelers from sub‐Saharan Africa 1. Its diagnosis was confirmed through examination of thin blood films and RDT. Although mixed‐*Plasmodium* species infection can occur in endemic areas where different malarial parasites overlap, they are rare in returned travelers 8. In this patient, RDT was initially suggestive of *P. falciparum* or mixed infection with *Plasmodium* non*‐falciparum*. The parasitemia was too low for a correct identification of the species. The PCR confirmed the *P. falciparum* infection, but other *Plasmodium* spp. PCR was not performed. The serology for *Plasmodium* spp. was not performed (patient living in a malaria‐endemic zone for several years and with previous episodes of malaria). Diagnosis of a mixed‐*Plasmodium* species infection was confirmed afterward when this patient was readmitted with a relapse of *Plasmodium spp*. infection (without having returned to an endemic zone). Relapse can occur due to the reactivation of *Plasmodium ovale* and *P. vivax* hypnozoites. In order to prevent relapse in cases like this, the eradication of Plasmodium latent forms with primaquine should be considered.

Our patient presented neurological symptoms on admission. An extensive investigation was performed, including lumbar puncture, brain MRI, and serology for other arthropod‐borne infections. The concomitant diagnosis of borreliosis was based on clinical presentation and positive serology for *Borrelia burgdorferi* sensu lato. Positive PCR for *B. burgdorferi* sensu lato in CSF also confirmed neuroborreliosis. Although antibody titers usually decline after antibiotic therapy, both IgG and IgM can persist positive for many months or even years after treatment 9.

There is no evidence in the literature of the influence of *Borrelia burgdorferi* infection in the course of malaria. However, some murine models suggest that co‐infection with malaria and relapsing fever (*Borrelia recurrentis* and *Borrelia duttonii)* can contribute to a more severe form of malarial infection, including mimicking cerebral malaria. This could be explained by an increased inflammatory response and endothelium dysfunction 10, 11.

The patient also presented pulmonary embolism as a manifestation of a primary antiphospholipid syndrome, which can be particularly severe in a patient with malaria due to a higher risk of thrombotic events. It should be also highlighted that patients with malaria frequently have severe thrombocytopenia and, for this reason, they do not start prophylactic heparin. The investigation performed showed positive lupus anticoagulant antibodies on two separate occasions, more than 12 weeks apart 12, 13. Frequently, the presence of autoantibodies is transiently found in association with some severe infectious disease, including antiphospholipid antibodies (anticardiolipin, anti‐beta 2 glycoprotein 1, and lupus anticoagulant), which might be induced by molecular mimicry phenomena and deregulation of the immune response during infection 14, 15. Apart from this, there is no standardized method of quantifying these autoantibodies and no calibration of the different kits, which can lead to a huge variability 16. Although the risk of thrombotic complications in this condition is usually low, whether patients develop antiphospholipid syndrome depends on both genetic and environmental factors 14. We consider this patient had an autolimited antiphospholipid syndrome associated with a severe infection as he had an unexplained thrombotic event with a favorable evolution with anticoagulation therapy and a persistently positive lupus anticoagulant.

To sum up, atypical malaria has a broad differential diagnosis, of which co‐infections represent a cornerstone. Making such diagnosis is of vital importance in terms of management and prognosis. This is particularly true in the case of the co‐infection of *B. burgdorferi*, due to the potentially devastating neurological and systemic manifestations and the therapeutic implications.

## Consent

Written informed consent was obtained from the patient for publication of this case report and any accompanying images. A copy of the written consent is available for review by the editor in chief of this journal.

## Authorship

NN: involved in general design and conception of the study and description of the first episode of hospitalization. ASP: involved in description of the second episode of hospitalization and reviewed the manuscript. HR: involved in description of the neurological disturbances and description of the neuroimaging. SS: reviewed the manuscript and substantially contributed to interpretation of clinical data. EP: described the antiphospholipid syndrome. AS: reviewed the manuscript. LS: reviewed the manuscript and contributed to the scientific content and final approval of the manuscript.

## Conflict of Interest

The authors declare no competing interests regarding the publication of this paper.
